# In Situ Cold-Junction Compensation Strategy for Semiconductor Thin-Film Thermocouples Based on a Pt Thin-Film Resistance Temperature Detector

**DOI:** 10.3390/s26144337

**Published:** 2026-07-08

**Authors:** Yuelong Li, Lantian Tang, Zhixuan Su, Yi Xu, Xianwei Qian, Yifan Wang, Qinnan Chen, Chao Wu

**Affiliations:** 1 Department of Mechanical and Electrical Engineering, Pen-Tung Sah Institute of Micro-Nano Science & Technology, Xiamen University, Xiamen 361005, China; liyuelong16163@163.com (Y.L.); 33520240157339@stu.xmu.edu.cn (L.T.); suzhixuan@stu.xmu.edu.cn (Z.S.); xianwei_qian@163.com (X.Q.); 34520232201159@stu.xmu.edu.cn (Y.W.); 2 Research Institute Aero-Engine, Beihang University, Beijing 100191, China; xuyi950@163.com; 3 AECC Sichuan Gas Turbine Research Establishment, Chengdu 610500, China; 4 Department of Mechanical Engineering, University of California at Berkeley, Berkeley, CA 94720, USA

**Keywords:** semiconductor thin-film thermocouple, ITO–In_2_O_3_, cold-junction compensation, Pt thin-film temperature detector, in situ temperature monitoring

## Abstract

Semiconductor thin-film thermocouples offer significant advantages for in situ temperature monitoring in advanced engineering equipment. However, the absence of a reliable cold-junction temperature compensation methodology has constrained their practical deployment. This study proposes an in situ cold-junction compensation strategy based on a Pt thin-film resistance temperature detector (RTD), wherein the Pt thin-film RTD is conformally integrated with an ITO–In_2_O_3_ thin-film thermocouple via sputtering and printing processes, enabling precise acquisition of cold-junction temperature without external temperature control apparatus. The fabricated Pt thin-film RTD exhibits a coefficient of determination of 0.99995 over the temperature range from ambient to 300 °C, with a temperature coefficient of resistance of 3810.09 ppm/°C, a maximum fitting error of merely 1.02 °C, repeatability precision superior to 1.46 °C, temperature resolution better than 0.2 °C, and a long-term drift rate as low as 0.006%/h. Under simulated practical operating scenarios, the RTD demonstrates superior thermal tracking performance relative to surface-mounted thermocouples. Conformal device fabrication is further realized on the curved surface of a turbine blade, where the RTD maintains characteristics consistent with those on planar substrates, and effective compensation up to 124 °C is achieved in butane flame thermal shock experiments. This strategy overcomes the limitation of conventional compensation methods to planar substrates, furnishing a reliable solution for high-precision in situ temperature monitoring on curved structures of hot-section components via semiconductor thin-film thermocouples.

## 1. Introduction

The iterative upgrading of advanced equipment has rendered the operating conditions of internal hot-end components increasingly severe [1,2,3]. Under the combined influence of extreme temperatures, high-velocity erosion, and stress fields, critical components are susceptible to failure phenomena such as fatigue damage and fracture [4,5,6]. Therefore, implementing health management and condition monitoring for hot-end components, alongside the accurate acquisition of key parameters such as the surface temperature of core parts, is strategically significant for optimizing performance, managing the entire life cycle, and enabling intelligent evolution [7,8,9].

Surface temperature measurement techniques for critical high-end components are currently an essential approach for intelligent condition monitoring and are broadly classified into contact and non-contact thermometry [10,11,12]. Contact thermometry includes sheathed thermocouples, temperature-indicating paints, and temperature-measuring rings [13,14]. These methods determine surface temperature by establishing thermal equilibrium through direct contact with the object. However, for surface temperature monitoring of critical components, contact sensors inevitably damage the component structure during installation, and the difference in heat capacity leads to low measurement accuracy, rendering such methods difficult to implement. Non-contact thermometry comprises infrared thermometry, fluorescence thermometry, and fiber-optic thermometry. Although non-contact methods do not require physical contact with the object, their measurement accuracy is affected by surface characteristics and is susceptible to environmental interference, which similarly limits their applicability for in situ temperature measurements on components [15,16,17].

High-temperature thin-film temperature sensors have attracted considerable attention in recent years owing to their advantages of high accuracy, rapid response, minimal disturbance to the flow field, and the capability for in situ fabrication [18,19,20]. They have emerged as a cutting-edge research direction for temperature perception in extreme environments [21,22,23]. According to their measurement principles, these sensors are categorized into thin-film thermocouples (TFTCs) and thin-film resistance temperature detectors (RTDs). Thin-film RTDs provide higher measurement accuracy but possess a relatively lower upper-temperature limit, which restricts their application in the ultimate harsh environments of advanced equipment [24,25]. In contrast, TFTCs exhibit greater temperature endurance. In particular, semiconductor-type TFTCs not only offer a high upper-temperature limit but also feature high sensitivity, enabling the resolution of minute temperature variations in harsh environments, and have thus become an advanced technology for in situ temperature measurement of critical components [26,27,28].

Among semiconductor-type devices, ITO/In_2_O_3_ TFTCs are the most extensively investigated. Current research focuses primarily on improving sensing performance at the material and process levels. Wang et al. enhanced the Seebeck coefficient of ITO/In_2_O_3_ TFTC by adjusting the sputtering pressure and oxygen content during deposition to modify the film microstructure and crystallization state [29]. Niu et al. employed pulsed laser deposition to fabricate an Al_2_O_3_ protective layer, enabling screen-printed ITO/In_2_O_3_ TFTCs to maintain a drift rate of 11.44 °C/h during long-term operation for 8 h at 1300 °C [30]. Liu et al. deposited an Al_2_O_3_ protective layer on the surface of Pt-PtRh TFTC via magnetron sputtering and compared the thermoelectric characteristics with and without encapsulation, demonstrating that the alumina layer effectively improved the high-temperature stability of the sensors [31]. Although these studies have significantly optimized the sensing performance of ITO-In_2_O_3_ TFTCs, the fundamental issue of effective cold-junction temperature compensation for semiconductor-type TFTCs has not been addressed.

The temperature measurement of TFTCs relies on the thermoelectric effect between the hot and cold junctions [30,32,33]. During operation under extreme conditions, the cold-junction temperature rises synchronously with the hot-junction temperature, making cold-junction temperature compensation a prerequisite for accurate measurement. Metal-type TFTCs typically employ compensation wires to extend the cold junction to a region with a relatively stable temperature, and use an ice-point bath or a water chiller to fix the cold-junction temperature at 0 °C or a constant value. However, due to the special nature of semiconductor TFTC materials, no reliable compensation wire is currently available to effectively mitigate the measurement errors caused by cold-junction temperature fluctuations [34]. The existing cold-junction compensation method for semiconductor-type TFTCs generally places the cold junction in a water-cooled device for temperature control and employs a discrete thermocouple to monitor the cold-junction temperature [35,36,37]. This approach is only suitable for TFTCs on flat substrates and is difficult to extend to in situ temperature monitoring of components with curved surface structures. To realize the widespread application of semiconductor-type TFTCs in high-precision temperature monitoring, it is necessary to develop a compensation method specifically for such devices, thereby creating prospects for their application in the field of high-precision temperature measurement in harsh environments.

To address this challenge, the present study proposes an in situ cold-junction compensation strategy for semiconductor TFTCs based on a platinum (Pt) RTD. Specifically, through the integration of sputtering and 3D printing processes, the Pt thin-film RTD is directly deposited onto the cold-junction of the ITO–In_2_O_3_ TFTC, thereby enabling precise acquisition of cold-junction temperature without reliance on external temperature control apparatus such as water-cooling systems. The comprehensive temperature sensing characteristics of the Pt thin-film RTD were systematically evaluated, and its compensation efficacy was examined under both calibration and simulated application conditions. Furthermore, the proposed scheme was validated on the surface of turbine blades, confirming its applicability to curved engineering components. This work aims to furnish a reliable and pragmatic compensation solution that eliminates the bottleneck of cold-junction temperature uncertainty, thereby facilitating the deployment of semiconductor TFTCs for high-precision in situ temperature monitoring in extreme environments.

## 2. Materials and Methods

### 2.1. Materials

In this study, two types of substrates were employed as carriers for validating the cold-junction compensation strategy of the thin-film thermocouple: Al_2_O_3_ substrates (Zhuhai Jiawei Ceramics Company Ltd., Zhuhai, China) and self-procured turbine blades. The ITO (In_2_O_3_:SnO_2_ = 90:10 wt%) and In_2_O_3_ target materials for the thin-film thermocouple were purchased from Beijing Jinyuan Advanced Materials Technology Company Ltd (Beijing, China). The cold-junction compensation Pt thin-film RTD was fabricated using platinum paste procured from Shenzhen Sryeo Electronic Paste Company Ltd (Shenzhen, China), while the protective layer was formed from glass–ceramic paste (1114 L) sourced from the same supplier. The lead wires consisted of 3 mm diameter Al_2_O_3_ wafers (Zhuhai Jiawei Ceramics Company Ltd., Zhuhai, China) and platinum wires, which were connected to the sensitive layer via commercial platinum paste (Shenzhen Sryeo Electronic Paste Company Ltd., Shenzhen, China). The turbine blades used in the application demonstration are made of GH2747 nickel-based alloy.

### 2.2. Fabrication Method

**Figure 1:**
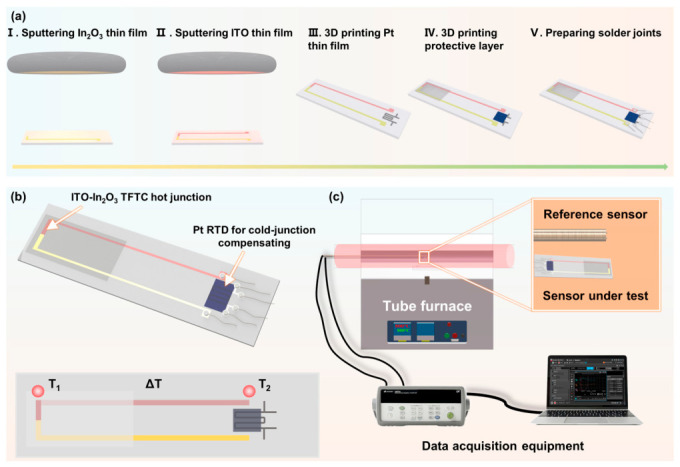
(**a**) Fabrication process of the ITO–In_2_O_3_ thin-film thermocouple integrated with a Pt thin-film RTD for cold-junction compensation. (**b**) Schematic diagram of the sensor structure. (**c**) Experimental setup for sensor testing.

The Al_2_O_3_ substrate was sequentially cleaned with alcohol, acetone, and deionized water, and then dried on a hot plate at 200 °C for 1 h. A stainless-steel mask was placed over the substrate, and ITO and In_2_O_3_ sensitive electrodes were sequentially deposited by magnetron sputtering at a base vacuum higher than 5 × 10^−4^ Pa, an operating pressure of 0.5 Pa, a power of 180 W, a substrate temperature of 200 °C, and an Ar flow rate of 80 sccm. After deposition, the sample was annealed at 550 °C for 2 h in a nitrogen atmosphere and then at 1100 °C for 1 h in air, with heating and cooling rates of 5 °C/min. Based on films prepared using the same process parameters in our previous work, the thicknesses of the sintered ITO and In_2_O_3_ films were approximately 4.12 and 4.23 μm respectively. A Pt thin film was then fabricated on the cold junction of the thermocouple by a printing process (printing voltage 8 V, displacement accuracy ±1 μm), followed by heating at 1100 °C for 1 h in air to form a conductive Pt network. Subsequently, a protective layer was deposited on the hot junction of the thermocouple and on the surface of the Pt thin-film RTD by printing and heated at 900 °C for 1 h in air, using the same heating and cooling rates. The ITO TFTCs were prepared by bonding platinum wires, Al_2_O_3_ wafers, and platinum paste, followed by sintering at 1100 °C for 30 min in air; these leads were arranged in a four-wire configuration to eliminate the influence of lead and contact resistance. The fabrication process is shown in Figure 1a. All heating processes were carried out in a muffle furnace.

### 2.3. Testing Principle

The fabricated sensor structure is shown in Figure 1b. The temperature measurement of the thin-film thermocouple relies on the thermoelectric effect. Accurate temperature measurement therefore requires precise knowledge of the cold-junction temperature, and this is precisely the role of the cold-junction Pt thin-film RTD. During measurement, the hot-junction temperature is denoted as T_1_, the cold-junction temperature as T_2_, and the temperature difference between them is

ΔT = T_1_ − T_2_ (1)

In practical applications, the temperature difference ΔT is derived from the output voltage signal of the thermocouple, and the hot-junction temperature T_1_ can be calculated by measuring the temperature of the Pt thin-film RTD T_2_. Its temperature is obtained by measuring its resistance:

R_T2_ = R_0_(1 + AT_2_ + BT_2_^2^) (2)

where R_T2_ and R_0_ represent the resistance values of the Pt thin-film RTD at T_2_ °C and 0 °C, respectively, and A and B are constants. The performance of the Pt thin-film RTD is determined by several key indicators, including resistance drift rate (DR), temperature coefficient of resistance (TCR, i.e., constant A), accuracy, long-term stability, and temperature resolution. The formula for the resistance drift rate is

DR = ΔR/(R_ref_ Δt) (3)

where R_ref_ denotes the initial resistance during the holding period, Δt represents the holding time, and ΔR is the change in resistance. During measurement, the hot junction of the TFTC was placed in the central constant-temperature zone of a tube furnace, while the cold junction was located in the low-temperature zone of the furnace (Figure 1c). An S-type thermocouple was positioned near the hot junction to provide a reference temperature, and a patch thermocouple was attached to the cold junction to provide a reference temperature for comparison with the Pt thin-film RTD.

In addition, the relationship between the thermoelectric response of the ITO/In_2_O_3_ thin-film thermocouple and the temperature difference can be described by the following equation:

U = A(ΔT)^3^ + B(ΔT)^2^ + C(ΔT) + D (4)

where U is the output voltage of the thin-film thermocouple, and A, B, C and D are constants. Once the above formula is established through calibration for the TFTC, in subsequent use, the temperature difference between the hot and cold junctions can be obtained by measuring the thermoelectric voltage of the TFTC. Combined with the temperature measured by the cold-junction Pt thin-film RTD, the accurate hot-junction temperature of the TFTC can be derived.

### 2.4. Characterization Methods

This study employed a scanning electron microscope (SEM; SUPRA55 SAPPHIRE, Carl Zeiss, Oberkochen, Germany) to observe the micromorphology. A stylus profilometer (Dektak XT, Bruker, Billerica, MA, USA) was employed to measure the film thicknesses.

### 2.5. Uncertainty Analysis

Following the Guide to the Expression of Uncertainty in Measurement (GUM), the combined standard uncertainty of the compensated hot-junction temperature was evaluated by propagating the uncertainties from the Pt RTD calibration (fitting residual 0.08 °C, reference thermocouple 0.30 °C, resistance measurement 0.017 °C, and long-term drift 0.029 °C) and the ITO–In_2_O_3_ TFTC calibration (fitting residual 0.12 °C, hot-junction reference thermocouple 0.75 °C, and voltage measurement 0.058 °C). The resulting combined standard uncertainty is u(T1) ≈ 0.83 °C. With a coverage factor k = 2, the expanded uncertainty is U(T1) = 1.66 °C (95% confidence level).

## 3. Results

### 3.1. Microstructural Characterization of the Cold-Junction Compensation Pt Thin-Film RTD

**Figure 2:**
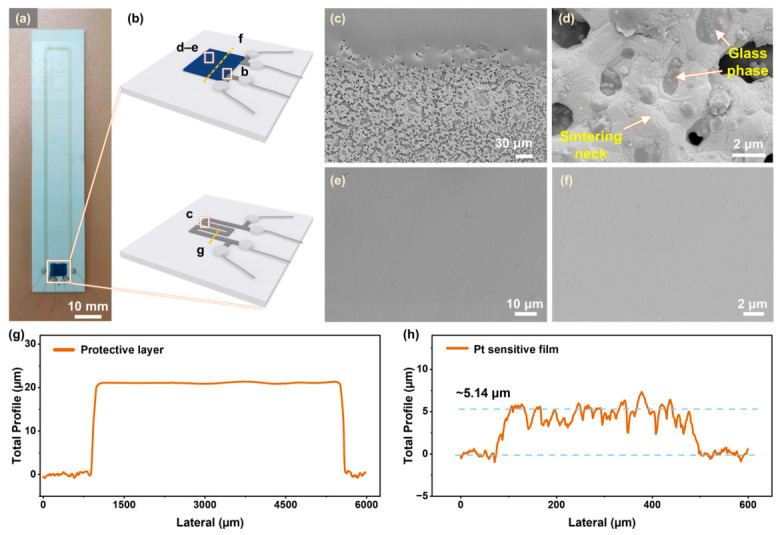
(**a**) Photograph of the ITO–In_2_O_3_ thin-film thermocouple integrated with a Pt thin-film RTD for cold-junction compensation. (**b**) Guide to the characterizations of Pt thin-film RTDs. (**c**) Transitional interface between the protective layer and the Pt thin film. (**d**) High-magnification SEM image of the Pt thin film. (**e**) SEM image of the protective layer. (**f**) High-magnification SEM image of the protective layer. (**g**) Cross-sectional profile of the protective layer. (**h**) Cross-sectional profile of the Pt film.

The stability of the microstructure is the foundation for the stable performance of thin-film sensors. Therefore, the microstructure of each layer of the compensating Pt thin-film RTD was examined using SEM. Figure 2a presents an optical image of the fabricated ITO-In_2_O_3_ TFTC together with its cold-junction compensation Pt thin-film RTD, which was integrated onto the same cold-junction region via a printing process.

Figure 2c shows an SEM image of the interface between the Pt sensitive film and the protective layer. The result reveals a clear interface without obvious interdiffusion and no harmful defects such as interfacial mismatch. Figure 2d displays the microstructure of the Pt thin film after annealing, where Pt particles developed sintering necks driven by the reduction in surface energy under high-temperature thermodynamic instability, with a glass phase embedded within them. This process markedly improved the thermal stability of the Pt thin film in high-temperature environments. Figure 2e,f show the micromorphology of the protective layer after annealing. Following high-temperature annealing, the glass powder melted sufficiently to form a dense microstructure without apparent pores or cracks. The dense protective layer effectively enhanced its erosion resistance at elevated temperatures and provided reliable shielding against corrosive species in extreme environments. Figure 2g shows that the protective layer has a smooth cross-section with a thickness of ~20.87 μm, whereas the unprotected Pt film is irregular with a thickness of ~5.14 μm. This indicates that although the protective layer fills the conductive network, its thickness does not significantly affect the blade surface flow field.

### 3.2. High-Temperature Performance of the Cold-Junction Compensation Pt Thin-Film RTD

**Figure 3:**
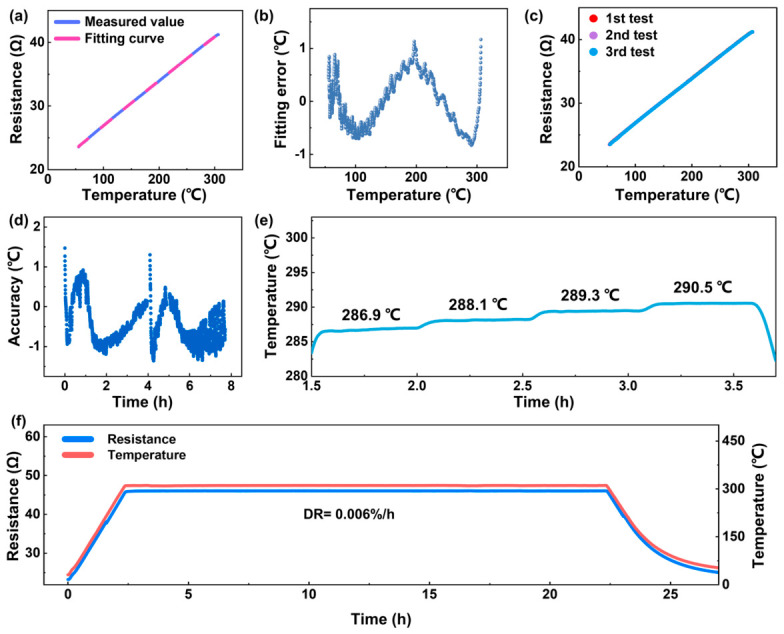
High-temperature test of the cold-junction compensation Pt thin-film RTD: (**a**) Temperature–resistance relationship. (**b**) Fitting error. (**c**) Repeatability test. (**d**) Accuracy test. (**e**) Resolution test. (**f**) Long-term stability test.

The sensing characteristics of the cold-junction compensation Pt thin-film RTD have a decisive influence on its compensation accuracy and long-term application. Therefore, a comprehensive investigation of its temperature sensing characteristics was carried out. Since the cold junction of the ITO/In_2_O_3_ thin-film thermocouple is typically located in a lower-temperature zone during actual deployment and is not directly exposed to extreme high-temperature environment, the investigation focused on the performance of the Pt thin-film RTD from room temperature to 300 °C. Figure 3a shows the calibration results of the Pt thin-film RTD within this temperature range. The relationship between its resistance and temperature can be fitted by the aforementioned formula, and the fitting results are listed in Table 1. The goodness of fit reached as high as 0.99995, and the temperature coefficient of resistance was 3810.09 ppm/°C, demonstrating a strong temperature dependence. This is an ideal characteristic for high-precision, high-resolution temperature sensors.

Furthermore, the full-range fitting error of the cold-junction compensation Pt thin-film RTD was analyzed (Figure 3b). The maximum fitting error was merely 1.02 °C, indicating that the error generated under this fitting model is negligible, which is favorable for subsequent high-precision in situ compensation. Figure 3c evaluates the repeatability of the Pt thin-film RTD. The three heating curves almost completely overlapped, reflecting its high repeatability. The specific accuracy data are shown in Figure 3d. The temperature measurement accuracy during the three cycles was better than 1.46 °C, a level of accuracy that ensures reliable monitoring of the cold-junction temperature. In addition, the resolution of a temperature sensor used for cold-junction compensation is also a key indicator. Figure 3e shows the test curves of the Pt thin-film RTD as the temperature was increased in steps of 1 °C. The temperatures of the standard thermocouple are annotated above the steps. Due to the temperature control accuracy of the tube furnace, the actual temperature rise steps were slightly larger than 1 °C. In all four step changes, the Pt thin-film RTD exhibited clear step responses, with deviations from the standard thermocouple not exceeding 0.2 °C. These results indicate that the RTD can rapidly respond to changes in the cold-junction temperature field in high-temperature environments, demonstrating high sensitivity and accuracy.

On the basis of the comprehensive performance evaluation of the Pt thin-film RTD, its long-term working capability was verified. It was held at 300 °C for 20 h to analyze its long-term stability, and the test results are shown in Figure 3f. The resistance drift was merely 0.006%/h, demonstrating its high stability in high-temperature environments. The long-term stability of its resistance under constant ambient temperature serves as the foundation for the aforementioned favorable performance of the Pt thin-film RTD. In practical operation, frequent recalibration is not required, thereby reducing maintenance costs and significantly improving the reliability of the sensing system.

### 3.3. Thermoelectric Response and Cold-Junction Compensation Effect

**Figure 4:**
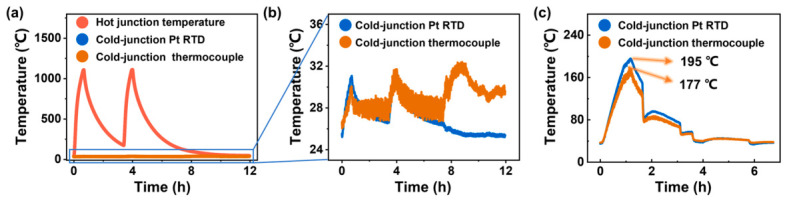
(**a**) High-temperature test signal of the cold-junction compensated thin-film thermocouple. (**b**) Signal comparison between the cold-junction compensation Pt thin-film RTD and the cold-junction compensation patch thermocouple under water-cooling condition. (**c**) Signal comparison between the cold-junction compensation Pt thin-film RTD and the cold-junction compensation patch thermocouple without water cooling.

On the basis of the verified favorable temperature measurement performance of the Pt thin-film RTD, its capability to compensate for the signal of the TFTC was investigated. Two types of tests were conducted: a calibration test with water cooling for cold-junction temperature control, and an application test without water cooling, to examine the ability of the Pt thin-film RTD to improve the calibration accuracy and the measurement accuracy of the TFTC, respectively. Under the water-cooled condition (Figure 4a), the center of the tube furnace provided the hot junction of the TFTC with two thermal cycles up to 1100 °C. In comparison, due to the existence of the water-cooling device, neither the cold-junction Pt thin-film RTD nor the patch thermocouple exhibited a visible temperature rise. A detailed examination of the signal (Figure 4b) reveals that, despite the minimal temperature increase, the Pt thin-film RTD exhibited a consistent response to the temperature fluctuations of the hot junction, which resulted from the combined effects of heat transfer from the rising hot-junction temperature and the cooling heat removal. In contrast, the patch thermocouple, owing to its larger thermal capacitance and inferior resolution, could hardly reflect the small-range temperature variation trend normally, and its temperature even failed to recover to the normal value after cooling, highlighting the advantage of the thin-film RTD for in situ accurate temperature measurement.

Subsequent tests were conducted under conditions that closely resemble actual operating conditions, i.e., without water cooling. The Pt thin-film RTD and the patch thermocouple exhibited similar temperature variation trends (Figure 4c). However, at the maximum temperature point, the measurement of the Pt thin-film RTD was 18 °C higher than that of the patch thermocouple. This phenomenon can be attributed to the fact that the patch thermocouple was bonded to the substrate with adhesive (thickness 0.3 mm), introducing a certain heat transfer process, and its discrete structure led to rapid heat exchange with the surrounding air, thereby resulting in a lower temperature reading. In contrast, the Pt thin-film RTD was tightly attached to the substrate with small self-heat capacity, and its temperature almost entirely reflected the true temperature.

### 3.4. High-Temperature Measurement Validation

**Figure 5:**
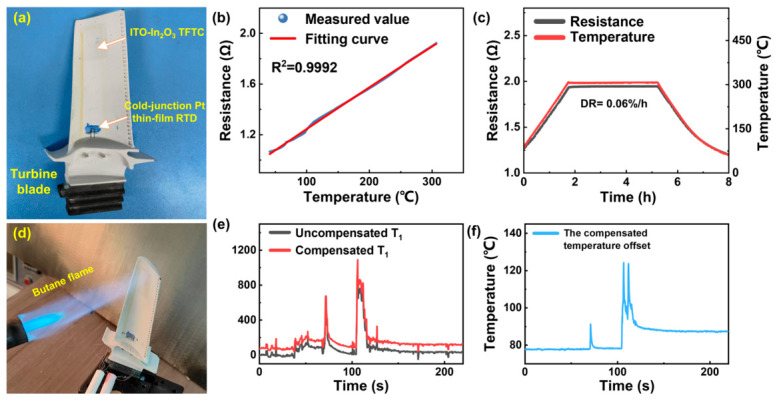
(**a**) Photograph of the cold-junction compensated thin-film thermocouple on the surface of a turbine blade. (**b**) Calibration of the R-T relationship of the cold-junction compensation Pt thin-film RTD. (**c**) Resistance stability test of the cold-junction compensation Pt thin-film RTD. (**d**) Photograph of the butane flame impingement test. (**e**) Comparison of thermocouple signals before and after cold-junction compensation under butane flame impingement. (**f**) Cold-junction compensated temperature of the thermocouple.

The aforementioned work demonstrated the advantages of the in situ Pt thin-film RTD in accurate compensation. Consequently, an ITO–In_2_O_3_ TFTC together with its cold-junction compensation RTD was further fabricated on the surface of a turbine blade. Figure 5a presents an optical image of the thin-film sensor on the turbine blade surface. The white insulating layer (NiCrAlY-YSZ-Al_2_O_3_ gradient composite coating) was fabricated via plasma spraying, with a thickness of 130–225 μm and a surface roughness Ra < 1.6 μm after grinding. The TFTC and its compensation RTD were conformally fabricated on the surface via sputtering and printing processes, exhibiting good adhesion. Figure 5b presents the calibration curve of the cold-junction compensation Pt thin-film RTD. Its goodness of fit was 0.9992, slightly lower than that of the device fabricated on a flat substrate. This is primarily due to the fact that the turbine blade possesses a larger heat capacity, leading to a greater temperature discrepancy between the standard thermocouple and the blade surface under conditions of temperature variation. Figure 5c shows the stability test curve of the Pt thin-film RTD on the turbine blade surface, which also demonstrated high stability at 300 °C, with a resistance drift rate of only 0.06%/h. This provides a solid foundation for accurate compensation in subsequent practical applications.

Furthermore, the hot junction of the thin-film thermocouple on the blade surface was subjected to a thermal shock test using a butane flame spray gun (Figure 5d) to simulate the severe thermal shock encountered under actual operating conditions. The butane flame torch was about 210 mm from the blade surface, with a flame temperature of about 1300 °C, and two shocks of 3 s and 8 s were applied. Figure 5e shows the output signals of the ITO–In_2_O_3_ TFTC before and after compensation. It is evident that the output signal trends before and after compensation are largely consistent; nevertheless, the compensated TFTC measurement is persistently higher than the uncompensated one. This arises from the output attenuation caused by heat transfer from the hot junction to the cold junction. To quantify the compensation effect of the Pt thin-film RTD, the compensation magnitude is displayed in Figure 5f. The peak compensation values correspond to the two maximum output signal values, indicating that the cold-junction compensation effect of the Pt thin-film RTD is more effective in more severe operating environments for the TFTC. The maximum compensation reached 124 °C. For in situ temperature monitoring under extreme operating conditions, such an error could lead to misjudgment in equipment health monitoring, highlighting the necessity of applying the cold-junction compensation thin-film RTD.

## 4. Conclusions

This study addresses the cold-junction compensation challenge inherent to semiconductor TFTCs by proposing an in situ compensation strategy based on a Pt thin-film RTD, which is conformally integrated with an ITO–In_2_O_3_ TFTC. The fabricated Pt thin-film RTD exhibits exceptional sensing performance over the temperature range from ambient to 300 °C, with a coefficient of determination of 0.99995, a TCR of 3810.09 ppm/°C, a maximum fitting error of merely 1.02 °C, repeatability precision superior to 1.46 °C, and temperature resolution better than 0.2 °C. The long-term drift rate at 300 °C is as low as 0.006%/h. Under both water-cooled and non-water-cooled conditions, the RTD accurately captures cold-junction temperature variations, demonstrating superior thermal tracking capability and resolution compared to surface-mounted thermocouples. Conformal device fabrication is further realized on the curved surface of a turbine blade, where the RTD maintains favorable drift rate of 0.06%/h. Butane flame thermal shock experiments demonstrate that the compensated TFTC readings are substantially enhanced, achieving a maximum compensation magnitude of 124 °C, thereby validating the effectiveness of this compensation strategy under extreme operating conditions. This approach eliminates the necessity for external temperature control apparatus and overcomes the limitation of conventional methods to planar substrates, furnishing a reliable solution for high-precision in situ temperature monitoring on curved structures of advanced engineering equipment.

## Figures and Tables

**Table 1:** Fitting results of the cold-junction compensation Pt thin-film RTD.

Equation	R_T_ = R_0_(1 + AT + BT^2^)
R_0_	19.50393 ± 0.00424
A	0.00381 ± 3.98293 × 10^−6^
B	−5.66799 × 10^−6^ ± 9.69185 × 10^−9^
R^2^	0.99995

## Data Availability

The data presented in this study are available on request from the corresponding author.
